# El Niño-Southern Oscillation affects the water relations of tree species in the Yucatan Peninsula, Mexico

**DOI:** 10.1038/s41598-021-89835-8

**Published:** 2021-05-17

**Authors:** Jorge Palomo-Kumul, Mirna Valdez-Hernández, Gerald A. Islebe, Manuel J. Cach-Pérez, José Luis Andrade

**Affiliations:** 1 El Colegio de la Frontera Sur Unidad Chetumal, Herbario, 77014 Chetumal, Q Roo México; 2 Departamento de Agricultura, Sociedad y Ambiente, CONACYT-El Colegio de la Frontera Sur Unidad Villahermosa, 86280 Villahermosa, TAB México; 3 Centro de Investigación Científica de Yucatán, Unidad de Recursos Naturales A.C., 97205 Mérida, YUC México

**Keywords:** Plant sciences, Ecology

## Abstract

We evaluated the effect of ENSO 2015/16 on the water relations of eight tree species in seasonally dry tropical forests of the Yucatan Peninsula, Mexico. The functional traits: wood density, relative water content in wood, xylem water potential and specific leaf area were recorded during the rainy season and compared in three consecutive years: 2015 (pre-ENSO conditions), 2016 (ENSO conditions) and 2017 (post-ENSO conditions). We analyzed tree size on the capacity to respond to water deficit, considering young and mature trees, and if this response is distinctive in species with different leaf patterns in seasonally dry tropical forests distributed along a precipitation gradient (700–1200 mm year^−1^). These traits showed a strong decrease in all species in response to water stress in 2016, mainly in the driest site. Deciduous species had lower wood density, higher predawn water potential and higher specific leaf area than evergreen species. In all cases, mature trees were more tolerant to drought. In the driest site, there was a significant reduction in water status, regardless of their leaf phenology, indicating that seasonally dry tropical forests are highly vulnerable to ENSO. Vulnerability of deciduous species is intensified in the driest areas and in the youngest trees.

## Introduction

Seasonally dry tropical forests have well-defined wet and dry periods within an annual cycle. In these forests, seasonal and inter-annual fluctuation in soil water availability could affect tree water balance^1,2^. The intensity and duration of the dry season may increase in regions affected by El Niño-Southern Oscillation (ENSO), which represents the main source of inter-annual climatic variation in most tropical regions and is reflected in a decrease or distribution of the amount of precipitation^3^. During an ENSO year, precipitation decreases significantly, mainly in the wet season of southeastern Mexico, in which the intra-summer drought, known locally as “*canícula*”^3,4^, intensifies. The mature phase of an ENSO event in southeastern Mexico and Central America is characterized by a considerable decrease in precipitation, with its highest peak between October and January^5^.

ENSO can cause intense and prolonged droughts throughout the subtropical region. ENSO in 2016 led to a reduction of up to 900 mm (34%) in annual precipitation in tropical forests of Panama^6^. This generates severe water stress in the plants, which in turn could lead to high plant mortality^6–9^. This mortality could differ by up to 34% among species^10^, and it has been noted that it occurs primarily in the larger trees^7^. Aboveground carbon stocks declined sharply during ENSO 2016 at a pantropical scale. Post-ENSO recovery was faster in drylands than in humid forests of Africa and America, which could be related to higher mortality^11^. Hence, high mortality rates could lead to changes in the composition and structure of plant communities in the long term^12^. Therefore, it is essential to understand the mechanisms that induce tree mortality during extreme droughts^9,13^. Several studies have found that functional traits associated with hydraulic safety margins are the main mechanisms that explain these mortality patterns^9,10,14,15^.

Among these functional traits, wood density is an indicator of tolerance to water deficit and generally has an inverse relationship with traits that prevent the negative effects of drought^16^. Wood density also relates to the wood relative water content and the water potential, which can indicate the water status of the plant^17,18^. Tolerance to low water potentials can be associated with high wood density and is linked to the hydraulic safety margin^14,19^. The specific leaf area involves a compromise between leaf longevity and resource capture capacity, and is a structural trait associated with drought tolerance. A high specific leaf area implies greater water loss and a low specific leaf area reduces water loss allowing adaptation to water stress^20^.

This adaptation has been linked to the patterns of leaf phenology. Deciduous species show high specific leaf area, while evergreen species need to maintain water transport to the canopy all year long and present sclerophyllous leaves^21,22^. Additionally, leaves of deciduous species have a high relative capacitance^23^ and leaves of evergreen species tolerate low water and osmotic potentials as a response mechanism to water stress^24,25^. Therefore, species with high wood density and low specific leaf area may be more resistant to drought-induced mortality^15^.

The Yucatan Peninsula, Mexico, is characterized by a precipitation gradient and a marked seasonal precipitation distribution. It is considered an extreme vulnerable area to ENSO^26,27^. Native forest tree species are adapted to the variability in precipitation and the *canícula*. Therefore the influence of ENSO on tree species would exhibit changes in their water status that can compromise their survival^3^. Future scenarios predict a precipitation reduction caused by climate change^28^, so the use of mathematical models fed with functional traits are necessary to understand the response of tropical tree species to droughts, including those caused by ENSO events^29^.

Thus, there is a need to generate information on the responses to drought of species at the plant community level to understand strategies that influence the survival of plant species^9,15^. Only few data on the effects of ENSO driven drought on tree species of seasonal dry tropical forests are available^10^. Most of these studies are large-scale meta-analyses^14^, so it is not possible to identify the mechanisms or strategies of the species at the local level^10^. Therefore, it is essential to generate studies with the largest number of functional traits collected simultaneously for a large number of tree species exposed to severe natural drought^30^. Hence, to fill important gaps of knowledge on how tree species react to dramatically changing climatic conditions, we address this complex interaction of drought and tree functional traits.

This study analyzes the impact of ENSO 2016 on the water status of eight tree species typical of a seasonally dry tropical forest of Yucatan Peninsula, based on a quasi-experimental field approach, which allows a more integrated understanding of which factors reduce or increase the response to ENSO. The factors considered are: years (2015 pre-ENSO, 2016 ENSO, 2017 post-ENSO), leaf patterns (four deciduous and four evergreen species), stem size (young: diameter at breast height ≤ 10 cm, mature: diameter at breast height ≥ 20 cm), and the precipitation gradient (mean annual precipitation dry site: 700 mm, intermediate site: 1000 mm, wet site: 1200 mm). We assumed that: (1) the influence of ENSO will be differential according to the leaf pattern. ENSO may have less impact on evergreen species, which commonly have a low specific leaf area and high wood density. These functional traits have been reported as keys to explaining species variations to drought response^15^. (2) The effect of ENSO on the water relations of trees may depend on the availability of water in the sites. Therefore, we predict that the level of water stress will be higher in the driest site than in the wettest sites. (3) The impact of ENSO on the water relations of trees may depend on the size of the individuals, and ENSO will affect more severely the water relations of the younger trees, due to their lower water storage capacity^10,31,32^. This knowledge is crucial for understanding how forests will respond to present and future changes in tropical precipitation regimes and modelling the patterns of survival and distribution of species in seasonally dry tropical forests to facilitate their management and conservation.

## Results

### Environmental characterization in the precipitation gradient

**Figure 1:**
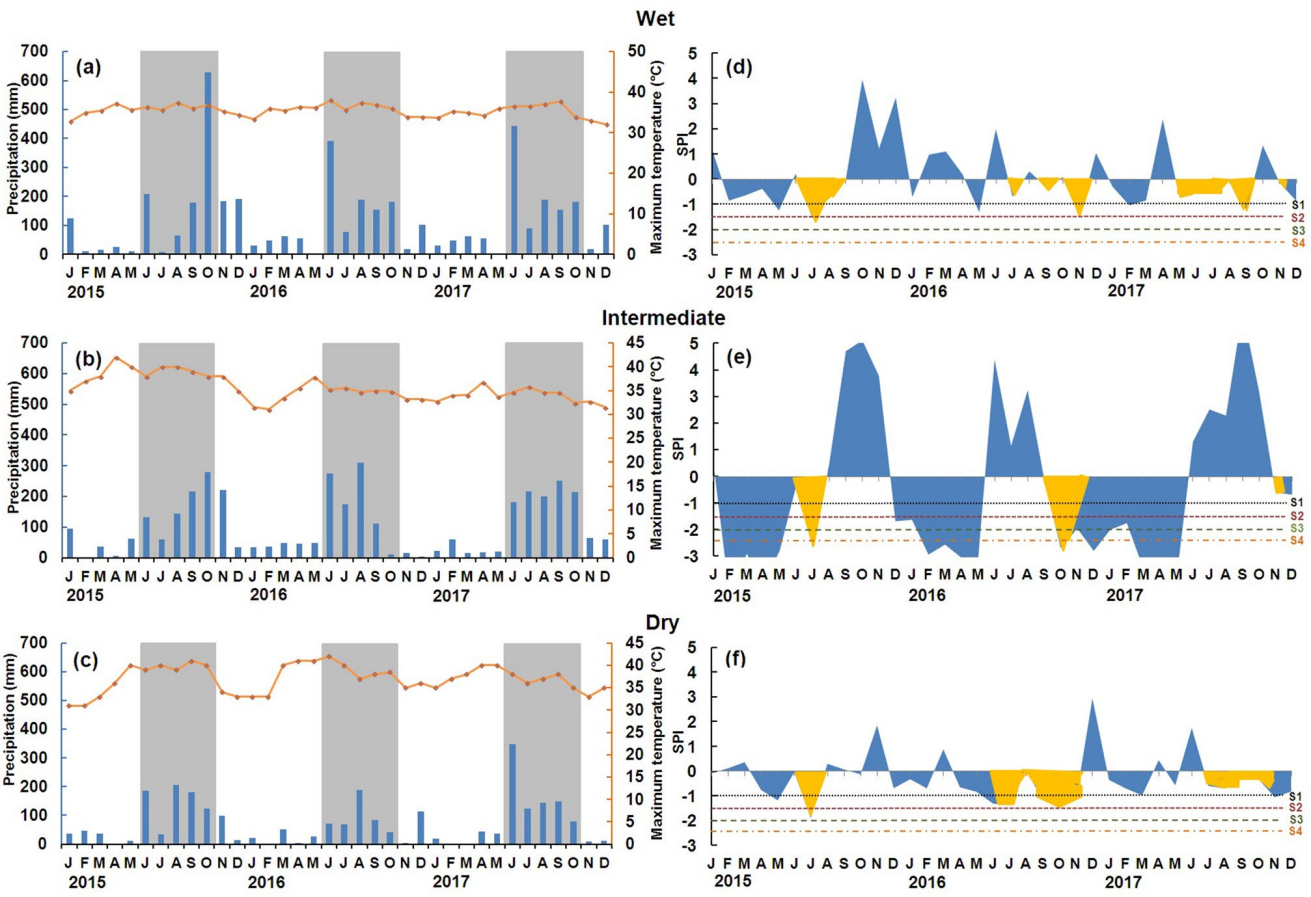
Environmental characterization recorded from 2015 to 2017 in the Yucatan Peninsula. Total annual precipitation and means of monthly temperature (**a**–**c**). The bars indicate precipitation, and the continuous line indicates temperature. The rainy season is indicated by grey shading. Monthly standardized precipitation index (**d**–**f**), Shaded in yellow indicates *canícula*. S1: mild drought, S2: moderate drought, S3: severe drought, S4: extreme drought.

Average annual temperature was similar among sites and among years (Fig. 1a–c). Even though the annual mean temperature was similar, the ENSO impacts on plants would differ with seasonal temperature variation^33,34^. Total annual precipitation was lower in 2016 at all three sites (Fig. 1a–c); the dry site had the lowest precipitation values at 654 mm and the highest number of dry months in 2016 (two to three more than the other two sites; Fig. 1a–c). The *canícula* occurred mainly in October and was the strongest during 2016 in all sites. The dry site had the longest *canícula* with 16 weeks (Fig. 1d–f). In addition, standardized precipitation index values indicate greater water stress intensity at the intermediate site (Fig. 1e).

### Effect of ENSO on water relations at sites with different precipitation

**Figure 2:**
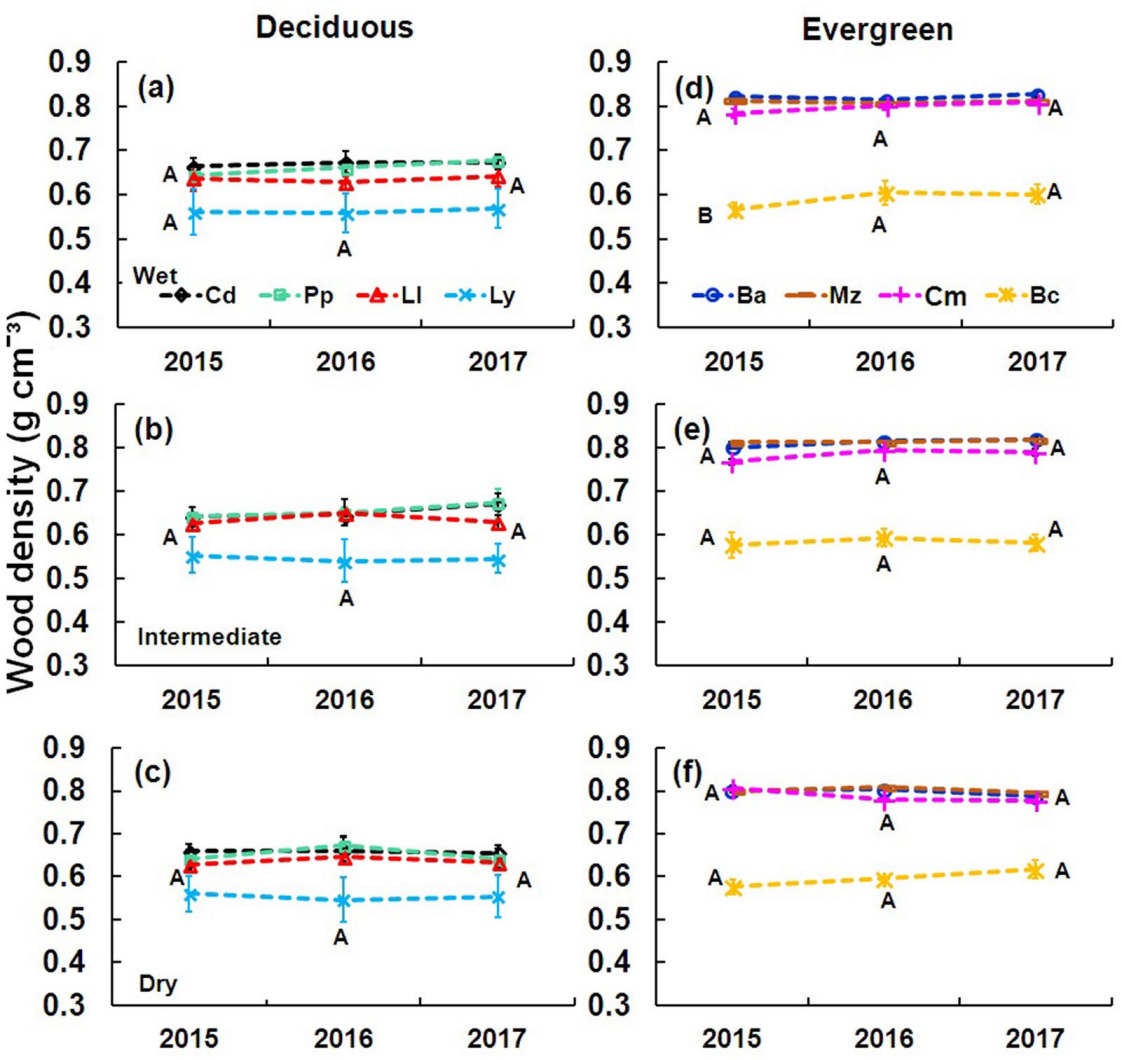
Wood density of deciduous and evergreen species from 2015 to 2017 considering young and mature diameter categories. Deciduous species (**a**–**c**): *Cordia dodecandra* (Cd), *Piscidia piscipula* (Pp), *Leucaena leucocephala* (Ll), *Lysiloma latisiliquum* (Ly). Evergreen species (**d**, **e**): *Brosimum alicastrum* (Ba), *Manilkara zapota* (Mz), *Chrysophyllum mexicanum* (Cm), *Byrsonima crassifolia* (Bc). Data are means ± SE. Capital letters next to the species indicate significant differences among years.

**Figure 3:**
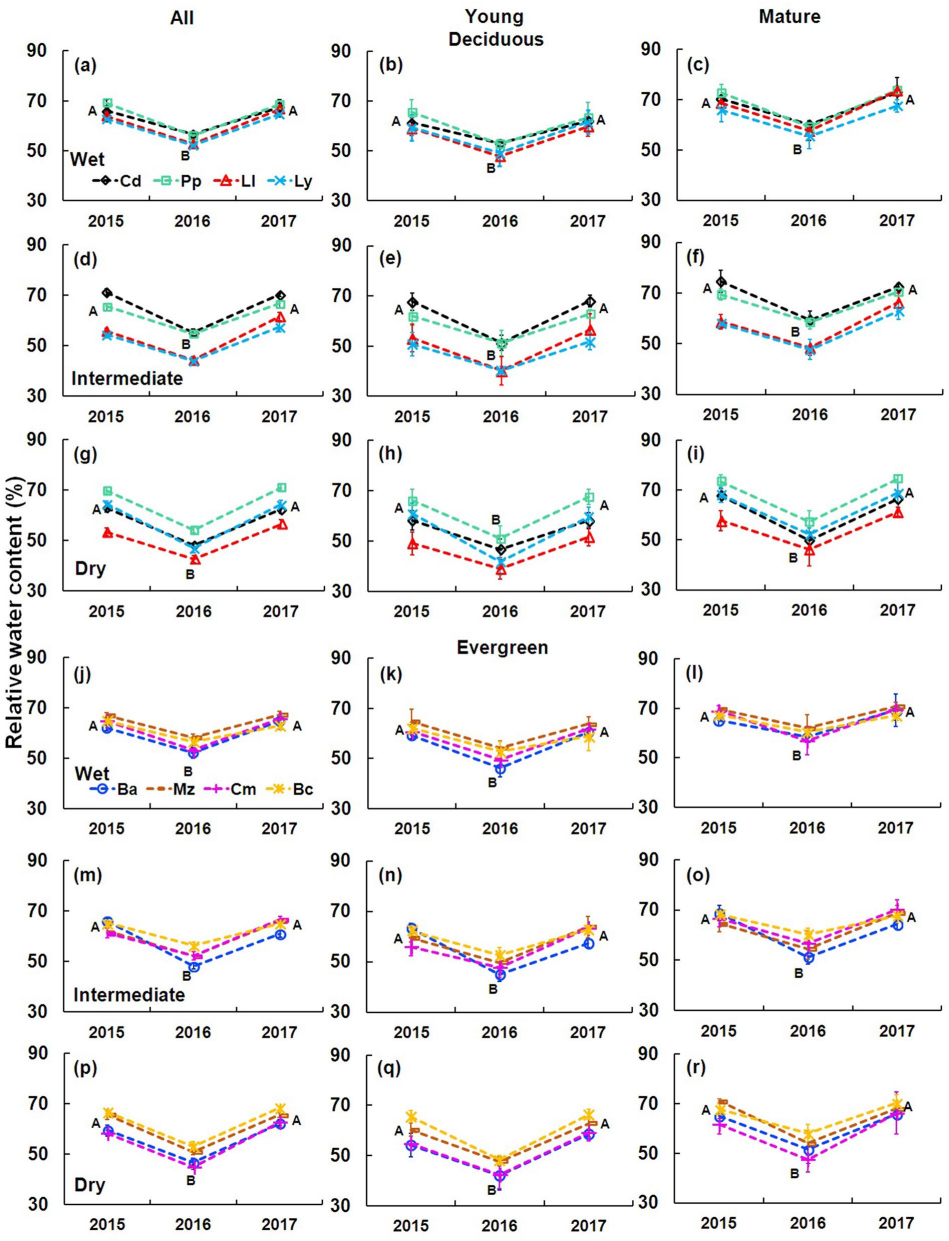
Relative water content of studied species in all sites from 2015 to 2017. Deciduous species (**a**–**i**): *Cordia dodecandra* (Cd), *Piscidia piscipula* (Pp), *Leucaena leucocephala* (Ll), *Lysiloma latisiliquum* (Ly). Evergreen species (**j**–**r**): *Brosimum alicastrum* (Ba), *Manilkara zapota* (Mz), *Chrysophyllum mexicanum* (Cm), *Byrsonima crassifolia* (Bc). Data are means ± SE. Capital letters next to the species indicate significant differences among years.

Evergreen species showed significantly higher wood density (0.75 ± 0.07 g cm^−3^; F = 268.4911, P < 0.0001; Table 1) than deciduous species (0.62 ± 0.08 g cm^−3^; Table 1). The lowest wood density was recorded in *Lysiloma latisiliquum* (deciduous; Fig. 2a–c) and *Byrsonima crassifolia* (evergreen; Fig. 2d–f). Wood relative water content was lowest during ENSO year (2016) for both deciduous and evergreen species (F = 125.1769, P < 0.0001; Table 1). Considering the sites and the leaf patterns, average wood relative water content was 7% higher in the wet site compared to the dry site (F = 13.2586, P < 0.0001; Table 1), but it was not significantly different between leaf patterns (F = 1.0422, P > 0.05; Table 1). For the eight species, the lowest relative water content in the deciduous species was recorded in *Leucaena leucocephala* (Fig. 3a–i) and in the evergreen species was for *Chrysophyllum mexicanum* (Fig. 3j–r).

**Table 1:** Functional traits of deciduous and evergreen species from 2015 to 2017.

Traits	All			Young			Mature		
	2015	2016	2017	2015	2016	2017	2015	2016	2017
**RWC (%)**									
W									
De	65.31 ± 3.07	54.30 ± 2.68	66.80 ± 3.61	61.18 ± 2.39	50.68 ± 2.03	61.76 ± 2.06	69.43 ± 2.17	57.91 ± 1.94	71.85 ± 3.03
Ev	64.79 ± 2.32	54.28 ± 2.86	65.10 ± 2.66	61.63 ± 1.98	50.61 ± 1.85	61.24 ± 1.71	67.94 ± 1.37	57.96 ± 2.50	68.97 ± 1.93
I									
De	61.30 ± 4.03	49.32 ± 3.71	63.50 ± 3.68	58.27 ± 3.84	45.76 ± 3.21	59.55 ± 3.39	64.53 ± 3.70	53.12 ± 3.33	67.71 ± 2.73
Ev	63.61 ± 2.61	52.20 ± 2.99	64.75 ± 2.44	60.24 ± 2.45	48.46 ± 2.44	61.77 ± 2.05	66.97 ± 1.42	55.94 ± 2.24	67.72 ± 1.85
D									
De	62.96 ± 4.16	47.97 ± 3.27	63.83 ± 3.74	58.54 ± 4.01	44.42 ± 2.71	60.02 ± 3.48	66.82 ± 3.33	51.36 ± 2.77	67.63 ± 3.02
Ev	62.36 ± 3.27	48.70 ± 3.32	64.49 ± 2.73	58.62 ± 3.14	44.78 ± 2.43	61.28 ± 2.45	65.82 ± 2.18	52.34 ± 3.02	67.49 ± 2.09
**Ψ**_**pd**_** (MPa)**									
W									
De	− 0.52 ± 0.06	− 0.69 ± 0.17	− 0.56 ± 0.07	− 0.52 ± 0.06	− 0.79 ± 0.08	− 0.59 ± 0.08	− 0.51 ± 0.05	− 0.71 ± 0.05	− 0.54 ± 0.05
Ev	− 0.65 ± 0.13	− 0.86 ± 0.12	− 0.62 ± 0.08	− 0.61 ± 0.14	− 0.83 ± 0.13	− 0.57 ± 0.10	− 0.70 ± 0.10	− 0.90 ± 0.10	− 0.05 ± 0.06
I									
De	− 0.52 ± 0.13	− 0.72 ± 0.13	− 0.49 ± 0.07	− 0.54 ± 0.15	− 0.74 ± 0.15	− 0.50 ± 0.07	− 0.51 ± 0.12	− 0.71 ± 0.12	− 0.47 ± 0.06
Ev	− 0.61 ± 0.17	− 0.86 ± 0.22	− 0.58 ± 0.14	− 0.60 ± 0.15	− 0.85 ± 0.20	− 0.58 ± 0.11	− 0.62 ± 0.20	− 0.86 ± 0.24	− 0.58 ± 0.16
D									
De	− 0.41 ± 0.05	− 0.72 ± 0.11	− 0.72 ± 0.07	− 0.37 ± 0.04	− 0.63 ± 0.10	− 0.39 ± 0.10	− 0.45 ± 0.05	− 0.82 ± 0.11	− 0.44 ± 0.04
Ev	− 0.46 ± 0.05	− 0.82 ± 0.13	− 0.43 ± 0.10	− 0.53 ± 0.04	− 0.81 ± 0.14	− 0.43 ± 0.08	− 0.40 ± 0.03	− 0.83 ± 0.11	− 0.43 ± 0.12
**Ψ**_**md**_** (MPa)**									
W									
De	− 1.02 ± 0.10	− 1.30 ± 0.13	− 0.99 ± 0.09	− 1.08 ± 0.10	− 1.32 ± 0.12	− 0.99 ± 0.10	− 0.96 ± 0.10	− 1.28 ± 0.15	− 0.98 ± 0.08
Ev	− 1.04 ± 0.10	− 1.34 ± 0.06	− 1..01 ± 0.06	− 1.02 ± 0.10	− 1.33 ± 0.06	− 1.01 ± 0.07	− 1.07 ± 0.10	− 1.36 ± 0.07	− 1.02 ± 0.07
I									
De	− 1.36 ± 0.21	− 1.63 ± 0.20	− 1.24 ± 0.20	− 1.34 ± 0.22	− 1.57 ± 0.20	− 1.19 ± 0.22	− 1.39 ± 0.22	− 1.70 ± 0.20	− 1.30 ± 0.17
Ev	− 1.30 ± 0.18	− 1.52 ± 0.18	− 1.17 ± 0.13	− 1.32 ± 0.12	− 1.53 ± 0.11	− 1.26 ± 0.06	− 1.27 ± 0.23	− 1.51 ± 0.24	− 1.09 ± 0.16
D									
De	− 1.18 ± 0.10	− 1.61 ± 0.16	− 1.16 ± 0.13	− 1.13 ± 0.12	− 1.60 ± 0.15	− 1.14 ± 0.13	− 1.22 ± 0.08	− 1.66 ± 0.18	− 1.19 ± 0.13
Ev	− 1.09 ± 0.23	− 1.51 ± 0.12	− 1.13 ± 0.12	− 1.17 ± 0.06	− 1.55 ± 0.10	− 1.25 ± 0.11	− 1.02 ± 0.32	− 1.47 ± 0.13	− 1.01 ± 0.10
**SLA (cm**^**2**^** g)**									
W									
De	154.7 ± 28.1	132.1 ± 26.2	155.8 ± 25.3	163.1 ± 27.1	127.6 ± 25.6	160.3 ± 29.7	156.6 ± 28.9	129.2 ± 26.7	158.6 ± 25.6
Ev	109.1 ± 6.4	104.2 ± 9.1	107.7 ± 5.6	107.4 ± 5.7	91.8 ± 4.8	109.2 ± 6.8	107.6 ± 6.2	90.9 ± 9.4	112.6 ± 4.6
I									
De	148.3 ± 28.4	130.6 ± 22.1	146.2 ± 25.7	163.9 ± 24.4	142.1 ± 19.3	160.5 ± 22.9	169.5 ± 31.7	145.5 ± 25.6	164.8 ± 27.9
Ev	100.2 ± 9.2	95.3 ± 8.7	104.1 ± 14.2	100.3 ± 8.1	85.1 ± 3.1	115.3 ± 18.9	115.1 ± 4.9	101.7 ± 7.1	119.1 ± 12.7
D									
De	139.5 ± 23.8	118.7 ± 17.4	137.4 ± 20.8	161.2 ± 23.5	134.7 ± 18.7	156.4 ± 19.7	151.5 ± 20.5	117.8 ± 15.5	147.2 ± 17.2
Ev	106.1 ± 7.4	93.9 ± 6.2	106.3 ± 9.4	110.4 ± 3.1	90.9 ± 5.3	116.5 ± 9.4	122.3 ± 5.1	93.6 ± 7.2	124.9 ± 5.2
**WD (g cm**^**−3**^**)**									
W									
De	0.62 ± 0.04	0.63 ± 0.03	0.64 ± 0.03	0.57 ± 0.04	0.58 ± 0.03	0.59 ± 0.04	0.67 ± 0.02	0.67 ± 0.03	0.68 ± 0.02
Ev	0.74 ± 0.05	0.75 ± 0.04	0.76 ± 0.05	0.74 ± 0.06	0.74 ± 0.05	0.75 ± 0.05	0.74 ± 0.04	0.76 ± 0.04	0.76 ± 0.04
I									
De	0.61 ± 0.03	0.62 ± 0.04	0.62 ± 0.04	0.56 ± 0.03	0.57 ± 0.04	0.58 ± 0.04	0.66 ± 0–02	0.67 ± 0.02	0.67 ± 0.03
Ev	0.73 ± 0.05	0.75 ± 0.05	0.75 ± 0.05	0.72 ± 0.05	0.74 ± 0.05	0.74 ± 0.05	0.75 ± 0.04	0.76 ± 0.04	0.77 ± 0.04
D									
De	0.62 ± 0.03	0.63 ± 0.04	0.62 ± 0.04	0.57 ± 0.03	0.58 ± 0.05	0.57 ± 0.04	0.66 ± 0.02	0.68 ± 0.02	0.66 ± 0.01
Ev	0.74 ± 0.05	0.75 ± 0.04	0.75 ± 0.03	0.73 ± 0.05	0.74 ± 0.04	0.73 ± 0.04	0.75 ± 0.04	0.75 ± 0.04	0.76 ± 0.03

Data are means ± SE.

*W* wet site, *I* intermediate site, *D* dry site, *De* deciduous, *Ev* evergreen.

**Figure 4:**
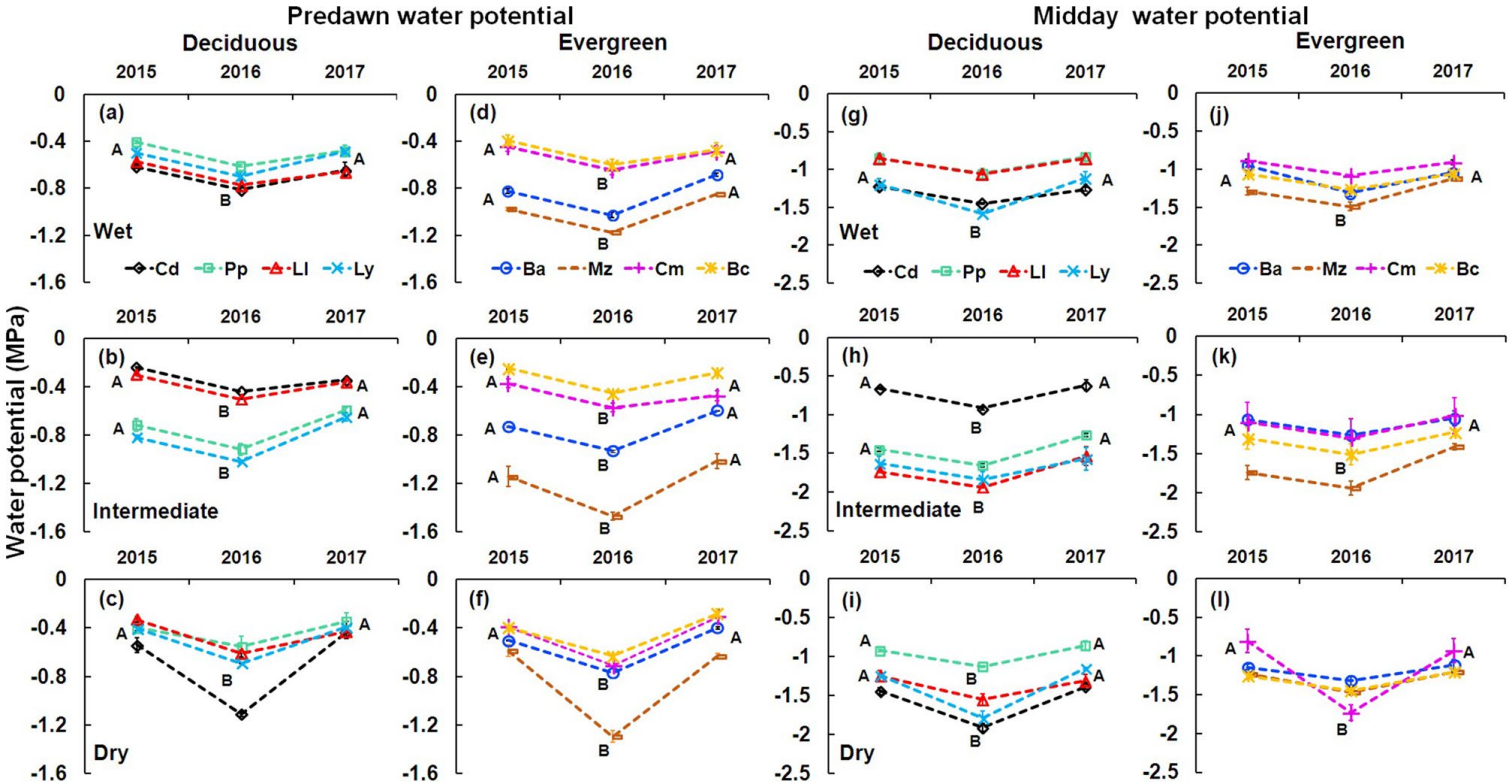
Predawn and Midday water potential for studied species in all sites from 2015 to 2017. Predawn water potential: panels are for deciduous species (**a**–**c**) and panels for evergreen species (**d**–**f**). Midday water potential: panels are for deciduous species (**g**–**i**) and for evergreen species (**j**–**l**). Deciduous species: *Cordia dodecandra* (Cd), *Piscidia piscipula* (Pp), *Leucaena leucocephala* (Ll), *Lysiloma latisiliquum* (Ly). Evergreen species: *Brosimum alicastrum* (Ba), *Manilkara zapota* (Mz), *Chrysophyllum mexicanum* (Cm), *Byrsonima crassifolia* (Bc). Data are means ± SE. Capital letters next to species indicate significant differences among years.

In all sites, the lowest xylem water potentials in both deciduous and evergreen species was recorded during ENSO (2016), for predawn (F = 56.7877, P < 0.0001; Table 1) and midday (F = 49.0454, P < 0.0001; Table 1). Predawn water potential was 0.10 MPa higher in deciduous species than in evergreen species (F = 20.5426, P < 0.0001; Table 1). This difference was also observed during the ENSO year (2016), where deciduous species exhibited values of predawn water potential of 0.14 MPa higher than evergreen species. Among species, all deciduous species had variable predawn water potentials among sites (Fig. 4a–c) and evergreen *Manilkara zapota* had the lowest predawn water potential in all sites (Fig. 4d–f), midday water potential between leaf patterns did not show differences (F = 2.4378, P > 0.05; Table 1). The lowest midday water potential was found in deciduous *Cordia dodecandra* and *Lysiloma latisiliquum* (Fig. 4g–i), and in evergreen *Manilkara zapota* and *Chrysophyllum mexicanum* (Fig. 4j–l).

**Figure 5:**
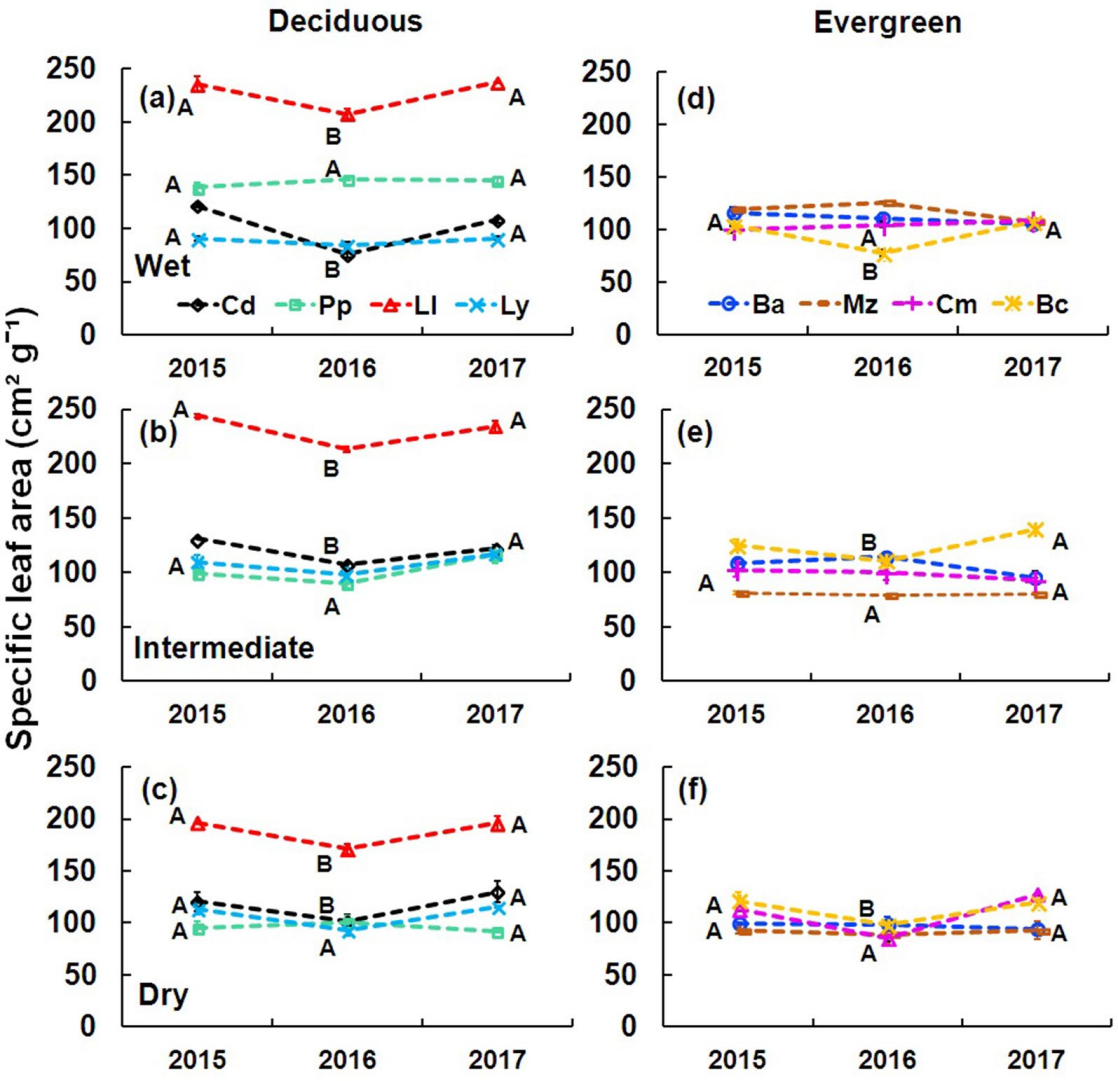
Specific leaf area for studied species in all sites from 2015 to 2017. Deciduous species (**a**–**c**): *Cordia dodecandra* (Cd), *Piscidia piscipula* (Pp), *Leucaena leucocephala* (Ll), *Lysiloma latisiliquum* (Ly). Evergreen species (**d**–**f**): *Brosimum alicastrum* (Ba), *Manilkara zapota* (Mz), *Chrysophyllum mexicanum* (Cm), *Byrsonima crassifolia* (Bc). Data are means ± SE. Capital letters next to species indicate significant differences among years.

Specific leaf area in deciduous and evergreen species was lower during the ENSO year (2016) than pre-ENSO and post-ENSO years (F = 3.1143, P < 0.05; Table 1; Fig. 5a–f), but no significant differences were found among sites (F = 2.9447, P > 0.05). Deciduous species showed higher specific leaf area values than evergreen species (F = 139.9195, P < 0.0001; Table 1), and *Leucaena leucocephala* showed the highest specific leaf area in all years and sites (F = 606.2844, P < 0.0001; Fig. 5a–c).

### Influence of ENSO on water relations considering diameter categories

Wood density was lower in the juvenile category (diameter at breast height < 10 cm, 0.66 g cm^−3^) than in the mature category (diameter at breast height > 20 cm, 0.72 g cm^−3^; F = 61.23, P < 0.0001; Table 1). Furthermore, evergreen species had a higher wood density than deciduous species in both size categories (young F = 191.7685, P < 0.0001; mature F = 114.2089, P < 0.0001; Table 1). Wood relative water content was 10% higher in mature individuals than in juveniles (F = 270.67, P < 0.0001) in all species (Table 1). Young individuals of deciduous and evergreen species had an average of 12–13% less relative water content in the ENSO year (2016) than in pre-ENSO and post-ENSO years (F = 79.7420, P < 0.0001; Table 1). Also, mature individuals of all species had a relative water content between 12 and 14% lower during the ENSO year (F = 104.9677, P < 0.0001; Table 1) than in the pre-ENSO and post-ENSO years. All individuals (young and mature) of evergreen species showed the least variation in the relative water content among species (Fig. 3j–r).

Predawn water potential was higher in mature than in young individuals of all species (F = 6.21, P < 0.05). Also, all species had the lowest predawn water potential values in 2016 (Table 1). Furthermore, deciduous species exhibited significantly higher values of predawn water potential than evergreen species (young F = 8.8589, P < 0.05; mature F = 11.6944, P < 0.001; Table 1).

For the midday water potential, no differences between diameter categories were found (F = 0.2618, P > 0.05; Table 1). Particularly, deciduous and evergreen species showed the lowest values in ENSO year (2016; Table 1). However, mature individuals of deciduous species had a higher midday water potential than evergreen species (F = 4.4993, P < 0.05; Table 1) for all years and sites.

Specific leaf area did not have significant differences between diameter categories (F = 0.0247, P > 0.05). In the pre-ENSO year, both young and mature individuals had the lowest average value (116.27 cm^2^ g^−1^; F = 94.889, P < 0.0001; Table 1). Furthermore, individuals of deciduous species had significantly higher specific leaf area values than those of evergreen species (young F = 81.1383, P < 0.0001; mature F = 56.6464, P < 0.0001; Table 1).

### Correlations between environmental and functional traits

In deciduous and evergreen species, a positive correlation of functional traits was found (predawn water potential, midday water potential, relative water content and specific leaf area) with precipitation, temperature and standardized precipitation index (Tables 2 and 3) and a negative correlation with pan evaporation (Tables 2 and 3). The standardized precipitation index exhibited a high correlation with water status parameters (predawn water potential, midday water potential and relative water content; Tables 2 and 3), which was stronger for relative water content in evergreen species (Table 2). The correlation among functional attributes showed that predawn water potential was the variable with the highest correlation with most attributes (Tables 2 and 3). In evergreen species, predawn water potential showed a positive relationship with midday water potential, relative water content, specific leaf area and a high negative correlation with wood density (Table 2). In deciduous species, predawn water potential was positively related to relative water content, midday water potential, and specific leaf area in descending order (Table 3).

**Table 2:** Correlation between environmental variables and functional traits of evergreen species.

Species	Functional traits		Environmental variables				Functional traits			
			Precipitation (mm)	Temperature (°C)	Evaporation (mm)	SPI	Ψ_pd_ (MPa)	Ψ_md_ (MPa)	RWC (%)	WD (g cm^3^)
*Brosimum alicastrum*	Ψ_pd_ (MPa)	Y	0.063	− 0.381*	− 0.441*	0.139				
		M	0.093	− 0.356*	− 0.441*	0.071				
	Ψ_md_ (MPa)	Y	0.684*	0.535*	0.091	0.627*	0.095			
		M	0.427*	− 0.127	− 0.295	0.579*	0.574*			
	RWC (%)	Y	0.531*	0.27	− 0.314	0.723*	0.46	0.691*		
		M	0.458*	0.261	− 0.349*	0.640*	0.493*	0.654*		
	SLA (cm^2^ g^−1^)	Y	0.302*	0.302	0.086	0.380*	− 0.274	0.275	0.239	
		M	0.181	0.361*	0.254	− 0.053	− 0.594*	− 0.354*	− 0.192	
	WD (g cm^3^)	Y	0.179	0.324	0.449*	0.225	− 0.292	0.169	0.084	0.215
		M	0.080	0.131	0.307	− 0.034	− 0.07	0.101	− 0.090	− 0.038
*Manilkara zapota*	Ψ_pd_ (MPa)	Y	0.203	− 0.106	− 0.299	0.325				
		M	0.091	0.045	− 0.15	0.126				
	Ψ_md_ (MPa)	Y	0.305	0.307	0.127	0.291	0.642*			
		M	0.048	0.081	0.193	− 0.062	0.813*			
	RWC (%)	Y	0.489*	0.222	− .0125	0.626*	0.658*	0.606*		
		M	0.434*	0.253	− 0.057	0.559*	0.529*	0.557*		
	SLA (cm^2^ g^−1^)	Y	0.311	0.513*	0.432*	0.068	0.169	0.434*	0.247	
		M	0.440*	0.710*	0.496*	0.323	0.109	0.331	0.312	
	WD (g cm^3^)	Y	0.406*	0.709*	0.593*	0.182	− 0.205	0.042	0.177	0.039
		M	− 0.001	− 0.101	− 0.044	0.080	− 0.332	− 0.261	− 0.101	− 0.244
*Chrysophyllum mexicanum*	Ψ_pd_ (MPa)	Y	0.478*	0.155	− 0.302	0.458*				
		M	0.262	− 0.132	− 0.607*	0.414*				
	Ψ_md_ (MPa)	Y	0.447*	0.485*	0.245	0.337	0.592*			
		M	0.23	− 0.032	− 0.299	0.308	0.621*			
	RWC (%)	Y	0.386*	0.108	− 0.069	0.553*	0.347*	0.347*		
		M	0.490*	0.198	− 0.117	0.654*	0.63*	0.623*		
	SLA (cm^2^ g^−1^)	Y	− 0.056	− 0.094	− 0.127	− 0.153	0.429*	0.498*	0.050	
		M	0.021	0.173	− 0.064	− 0.023	0.408*	0.215	0.248	
	WD (g cm^3^)	Y	− 0.081	− 0.227	− 0.352	− 0.15	0.11	0.376*	0.122	0.035
		M	− 0.328	0.094	0.27	− 0.215	− 0.132	0.156	0.017	0.060
*Byrsonima crassifolia*	Ψ_pd_ (MPa)	Y	0.446*	− 0.091	− 0.308*	0.615*				
		M	0.18	− 0.381*	− 0.731*	0.443*				
	Ψ_md_ (MPa)	Y	0.494*	0.381*	− 0.023	0.553*	0.541*			
		M	0.363*	0.357*	0.195	0.389*	− 0.132			
	RWC (%)	Y	0.383*	0.004	− 0.487*	0.512*	0.696*	0.476*		
		M	0.351*	− 0.064	− 0.433*	0.473*	0.604*	0.364*		
	SLA (cm^2^ g^−1^)	Y	0.093	− 0.241	− 0.273	0.412*	0.436*	0.286	0.521*	
		M	0.064	− 0.541*	− 0.508*	0.161	0.67*	0.078	0.49*	
	WD (g cm^3^)	Y	− 0.352*	− 0.216	0.214	− 0.379*	− 0.296	− 0.209	− 0.183	− 0.137
		M	− 0.292	− 0.005	0.12	− 0.165	− 0.099	− 0.186	− 0.132	− 0.146

SPI: standardized precipitation index (the value corresponds to the month of October). Pearson correlation, the values marked with asterisks are significant (P < 0.005).

**Table 3:** Correlation between environmental variables and functional traits of deciduous species.

Species	Functional traits		Environmental variables				Functional traits			
			Precipitation(mm)	Temperature (°C)	Evaporation (mm)	SPI	Ψ_pd_ (MPa)	Ψ_md_ (MPa)	RWC (%)	WD (g cm^3^)
*Piscidia piscipula*	Ψ_pd_ (MPa)	Y	0.121	− 0.008	− 0.071	0.021				
		M	0.479*	0.187	− 0.172	0.442*				
	Ψ_md_ (MPa)	Y	0.225	0.32	0.193	0.119	0.761*			
		M	0.258	0.212	0.068	0.144	0.760*			
	RWC (%)	Y	0.408*	− 0.085	− 0.351*	0.481*	0.464*	0.434*		
		M	0.426*	− 0.152	− 0.290	0.498*	0.695*	0.475*		
	SLA (cm^2^ g^−1^)	Y	0.38*	0.760*	0.612*	0.188	0.123	0.517*	− 0.027	
		M	0.333*	0.603*	0.641*	0.245	0.294	0.347*	0.108	
	WD (g cm^3^)	Y	− 0.094	0.134	0.321	0.036	0.067	0.012	0.003	0.225
		M	− 0.263	− 0.173	0.175	− 0.151	− 0.156	− 0.219	− 0.181	0.090
*Cordia dodecandra*	Ψ_pd_ (MPa)	Y	0.042	− 0.015	− 0.251	0.194				
		M	0.222	− 0.049	− 0.343	0.430*				
	Ψ_md_ (MPa)	Y	0.144	− 0.124	− 0.281	0.487*	0.349*			
		M	0.337	0.0124	− 0.227	0.608*	0.761*			
	RWC (%)	Y	0.415*	− 0.114	− 0.380*	0.738*	0.106	0.628*		
		M	0.488*	0.341	− 0.19	0.697*	0.776*	0.674*		
	SLA (cm^2^ g^−1^)	Y	0.208	− 0.339	− 0.648*	0.373*	0.203	0.53*	0.447*	
		M	0.272	− 0.223	− 0.607*	0.449*	0.643*	0.644*	0.551*	
	WD (g cm^3^)	Y	0.058	0.214	0.263	− 0.016	− 0.029	− 0.132	0.028	− 0.202
		M	− 0.108	− 0.034	0.192	0.0221	− 0.194	− 0.078	− 0.075	− 0.057
*Leucaena leucocephala*	Ψ_pd_ (MPa)	Y	0.021	− 0.444*	− 0.835*	0.243				
		M	0.28	− 0.144	− 0.677*	0.474*				
	Ψ_md_ (MPa)	Y	0.352*	0.534*	0.307	0.152	0.408*			
		M	0.455*	0.593*	0.472*	0.332*	− 0.282			
	RWC (%)	Y	0.546*	0.380*	− 0.032	0.684*	0.045	0.449*		
		M	0.458*	0.378*	0.176	0.568*	0.152	0.649*		
	SLA (cm^2^ g^−1^)	Y	0.662*	0.581*	0.101	0.652*	− 0.076	0.471*	0.727*	
		M	0.550*	0.419*	0.057	0.670*	0.490*	0.286	0.540*	
	WD (g cm^3^)	Y	− 0.257	− 191	0.091	− 0.3	− 0.0322	− 0.301	− 0.166	− 0.17
		M	0.132	0.103	0.168	0.030	− 0.127	0.134	0.026	− 0.055
*Lysiloma. latisiliquum*	Ψ_pd_ (MPa)	Y	0.236	− 0.042	− 0.102	0.132				
		M	0.181	0.120	− 0.116	0.208				
	Ψ_md_ (MPa)	Y	0.312	0.141	− 0.163	0.344*	0.523*			
		M	0.476*	0.417*	0.050	0.338*	0.751*			
	RWC (%)	Y	0.396*	0.332*	0.102	0.374*	0.636*	0.739*		
		M	0.321	0.161	− 0.173	0.371*	0.710*	0.796*		
	SLA (cm^2^ g^−1^)	Y	− 0.128	− 0.138	− 0.238	0.218	0.222	0.322	0.296	
		M	− 0.103	− 0.522*	− 0.618*	0.076	0.421*	0.533	0.376*	
	WD (g cm^3^)	Y	0.122	0.211	0.002	0.238	0.046	0.343*	0.361*	0.029
		M	0.161	0.033	0.067	0.060	0.24	0.062	− 0.079	− 0.014

SPI: standardized precipitation index (the value corresponds to the month of October). Pearson correlation, the values marked with asterisks are significant (P < 0.005).

In evergreen species, correlations were higher for the young than for the mature individuals, mainly for midday water potential and precipitation (young = 0.429, mature = 0.172; Table 2). In deciduous species, mature individuals showed higher correlation values than younger individuals for predawn water potential and precipitation, specific leaf area and standardized precipitation index (Table 3). The correlation between functional attributes showed that predawn water potential was the variable with the highest correlation with most attributes (Tables 2 and 3).

## Discussion

The analyzed tree species of the Yucatan Peninsula showed a reduction in wood relative water content, water potential and specific leaf area during the intense drought caused by ENSO 2016. This behavior was stronger in deciduous than in evergreen species. In addition, due to their larger size, mature individuals were less vulnerable to water deficit, due to their higher stem water storage, reflected in 15% more wood relative water content, compared to young individuals.

ENSO of 2016 was registered by NOAA^4^ as one of the most intense droughts, and due to the magnitude and impact of this ENSO, precipitation anomalies were reported globally. In the western Pacific a positive anomaly was recorded, which was caused by the influence of the ENSO event in association with a nonlinear southern humidity advention^35,36^. Recently, studies suggest that the coastal ENSO of 2017 presented severe impacts in the eastern Pacific region, due to an asymmetric double band of the intertropical convergence zone (ITCZ) along the equator and due to the association with positive feedback between coastal warming, deep atmospheric convection and coastal winds, which generated positive anomalies in large-scale precipitation^37–39^. On the other hand, in Central America and the Yucatan Peninsula a strong negative anomaly in precipitation has been recorded. So, the *canícula* of that year was stronger and its effect intensified in the study site with less precipitation^27^.

The water limitation induced by the decrease and distribution of precipitation during the ENSO 2016 was remarkable in the driest site, with 34% less precipitation than the lowest historical value and an extended dry season (10 months). The mega-drought of 2015–2016 generated an increase in the vapor pressure deficit which intensified the demand for water exchange in the forests, particularly during the dry season^40^. Likewise, the less negative standardized precipitation index values in the intermediate site indicate that 2016 was the driest of the analyzed 3 years, and even drier than values reported in the dry site. Therefore, the impact on individuals of the intermediate site could have been greater than for individuals from the two other sites. However, adaptation to climatic uncertainties caused by water variability can be determined by genetic responses^3^, considering that the study area has been influenced by ENSO for thousands of years^26^.

The lowest relative water content was recorded in ENSO conditions (2016) in all sites. The relative water content reduction matched with lower xylem water potentials. Xylem water potentials have been considered a sensitive indicator of the water status of plants^6^, as this has been observed in sites with adverse micro-environmental conditions^41,42^. Additionally, in 2016, two evergreen species (*Byrsonima crassifolia* and *Chrysophyllum mexicanum*; Fig. 5f) and three deciduous species (*Cordia dodecandra, Leucaena leucocephala,* and *Lysiloma latisiliquum*; Fig. 5c) reduced their specific leaf area. Water deficit in the ENSO year affected the specific leaf area, a trait very sensitive to variations in soil moisture^43^.

Although local species are adapted to the *canícula*, the stronger intensity and duration of the water deficit in the dry site generated a stronger effect on relative water content and xylem water potentials of individuals. At this forest site, evergreen species exhibited lower values of predawn water potentials than deciduous species. This could have important consequences on water availability affecting forest productivity^3^. Evergreen *Manilka zapota* exhibited a high wood density (0.80 g cm^−3^)*,* and was one of the species with the lowest predawn water potentials and midday water potentials in all sites, especially in the dry site, which can confirm evergreen species having a strong tolerance to low water potentials^44^. The high tension in the water-carrying system can be buffered by the presence of a high wood density and higher density of small vessels^17,45^. Therefore, these species are more resistant to suffering hydraulic failure and surviving drought due to greater tolerance to desiccation^46^.

The sharp decline of relative water content in the dry site during ENSO 2016, compared to 2015 and 2017, evidenced the capacity of species to tolerate drought. Our data show that a 20% reduction in precipitation recorded at the wet and intermediate sites led to a 10% decrease in RWC for all species. Whereas, a 30% decrease in precipitation in the driest site induced a 14% reduction in RWC in all species. This response indicates that species exhibited plasticity in drought tolerance related traits at intraspecific and interspecific levels which would favor tolerance to the extreme drought of ENSO 2016. However, in spite of this tolerance to water stress, long-term droughts or very intense *canícula* can have a strong effect on the physiology, even compromising species survival. A 50% reduction in precipitation can lead to an increase in tree mortality due to a strong link between drought vulnerability and hydraulic safety margins^10^.

This decrease was also observed in the midday water potential. In the ENSO year, water potential values of − 1.6 MPa were recorded, while in the pre-ENSO and post-ENSO year values were higher (− 1.18, and − 1.16 MPa, respectively; Table1). Under high water stress conditions, the control of water potential is common and tree species can tolerate relatively low osmotic potentials (− 1.88 MPa^47^). The deciduous legumes (*Lysiloma latisiliquum, Leucaena leucocephala* and *Piscidia piscipula*) have abundant storage parenchyma (unpublished data), allowing an intracellular water reserve and dampening water stress^17^. This is a distinctive feature in tree species of seasonally dry tropical forests^48^, mainly in legumes species^49^. Likewise, the abundant presence of parenchyma in some legumes allows maintaining high water conductance. Additionally, legumes have a high density of small vessels facilitating their hydraulic safety and exhibit less vulnerability in the xylem and therefore greater tolerance to drought^45^.

In all study sites, a reduction in specific leaf area combined with a reduction in relative water content for some deciduous species in 2016 was observed. Leaves developed under water-stressed conditions generally have a lower specific leaf area than leaves produced in higher precipitation sites^43^ due to variations in leaf thickness or density^50^. Leaves with a high density are less susceptible to drought, as they are more resistant to water loss^51^. Variations in leaf structure in response to water limitation can generate elastic changes in cell tissues^52^.

Low specific leaf area values and little inter-annual variation in evergreen species (mainly *Brosimum alicastrum* and *Manilkara zapota*; Fig. 5d–f) indicate desiccation tolerance, which is characteristic for reducing herbivory damage and increasing leaf longevity, allowing longer carbon gain^53^. It would be challenging to study these species in common garden experiments, Mayan back yards, or even urban parks because it has been found that certain tree species can respond to the water deficit through a change in the reallocation of resources, investing less in foliage and more in fructification^5^.

The mature category of trees showed higher relative water content and higher wood density than younger trees, remarking the importance of stem size in water storage capacity^54^. This would indicate that the mature category might be less vulnerable for its increased water storage capacity. It has been reported that water stored in large trees provides up to 30% of the daily sap flow, allowing transpiration for up to 1 week^32^. In addition, in tropical forests of Panama, the capacity of water storage in the stem in tree species is directly related to the size of the sapwood and the height of the tree^31,41^. In the Yucatan Peninsula, this relationship of water storage with stem diameter is positively correlated in tree species of the family Ebenaceae, Malvaceae, Sapotaceae and Burseraceae^55^.

The evergreen species *Manilkara zapota* and *Brosimum alicastrum* showed the smallest relative water content difference between categories, 7% and 6% respectively. Similarly, wood density values between categories were also very close, suggesting a similar morpho-physiological response in water storage in the stem^56^. Wood density has been related to tolerance to low osmotic potential^57^, high capacitance, and water storage in the stem^23^. Additionally, one distinctive feature of evergreen species is that they have extensive roots that allow them to explore a larger volume of deep water in the soil^58^, which also relates to a high-water storage in the stem^31^.

The similarity in leaf water potential between diameter categories can be explained to the higher foliar coverage accompanying the larger trees, which required higher water content to supply the diurnal evaporative demand^41^. This is also related to the higher number of species with a high positive correlation between xylem water potentials and relative water content in the mature category. Most likely, younger trees had strong regulatory responses to drought caused by ENSO, whereas trees with larger stems are not compromised during drought, possibly to greater exploration of water by roots to deeper soil^6^. Also, the water stored in the stem in larger trees promotes the efficient water transport^59^. However, the exposure to high solar radiation and high vapor pressure deficits can affect stem hydraulic safety and leaf temperature regulation^60^. Therefore, while larger trees may have a root system that explores a larger volume of soil, it may not be enough to maintain an adequate water balance if drought increases. These response strategies often differ in species with different leaf patterns, with greater water transport efficiency in deciduous species^61^ and less vulnerability in xylem in evergreen species^43^. However, a convergence in hydraulic traits has also been found between deciduous and evergreen species^62^.

The studied tree species responded to ENSO 2016, by decreasing the wood relative water content, lower water potential and a reduction in the specific leaf area. The decrease in relative water content and water potential was stronger in the dry site and was more evident in deciduous than in evergreen species. Some of the most affected species were *Cordia dodecandra, Leucaena leucocephala* and *Lysiloma latisiliquum,* which decreased specific leaf area in response to water availability. This decreases the probability of physical damage to leaves by desiccation. In contrast, evergreen species, mainly *Manilkara zapota* and *Brosimum alicastrum* tolerate lower predawn water potentials than deciduous species. This indicates diverse responses to drought within and among different functional groups.

Trees of the mature category have an advantage, because of their higher stem water storage capacity. Therefore, in a severe drought, mature trees could possibly respond more effectively compared to younger trees. This study indicates that seasonally dry tropical forests are highly vulnerable to ENSO effects, and vulnerability is enhanced in drier regions and in the younger tree individuals. To fully understand eco-physiological responses to extreme climate events we need in situ studies of functional traits variability of many tropical dry seasonal forest species. Forthcoming studies should focus on hydraulic architecture, considering wood anatomical traits, and cavitation vulnerability curves, among others, for several tree species in different scenarios. Since, the mechanisms associated with hydraulic safety margins can help understand species plasticity, considering the hydroclimate variability faced by dry seasonal tropical forests.

## Materials and methods

### Study area

Three seasonally dry tropical forests along a precipitation gradient (700–1,200 mm year^−1^; Supplementary Fig. 1) were considered. Dry: Dzibilchaltún National Park, located at north of Yucatan; Mexico, between 21° 05′ N and 89° 99′ W. This area has an average annual temperature of 25.8 °C and an annual precipitation of 700 mm, with the highest precipitation occurring between June and October^63^. Intermediate: X-pichil, a forest reserve located between 19° 41′ N and 88° 22′ W, has an average annual temperature of 26.4 °C and an annual precipitation of 1000 mm, with the highest precipitation occurring between June and October^64^. Wet: Chetumal, located between 18° 32′ N and 88° 15′ W, has an average annual temperature of 27 °C, and an annual precipitation of 1200 mm, the highest precipitation is between May and October^65,66^.

### Studied species

The studied tree species were chosen according to their distribution (occurring in the three sites), and their contrasting phenological patterns (evergreen and deciduous). The evergreen species were: *Brosimum alicastrum* Sw., *Manilkara zapota* (L.) P. Royen, *Chrysophyllum mexicanum* Brandage ex Standl, *Byrsonima crassifolia* (L.) Kunth. The deciduous species were: *Cordia dodecandra* DC., *Piscidia piscipula* (L.) Sarg., *Lysiloma latisiliquum* (L.) Benth., *Leucaena leucocephala* (Lam.) de Wit.

In each species, two stem diameter categories (diameter at breast height: young ≤ 10 cm and mature ≥ 20 cm) were considered. In all cases we selected four individuals of similar size, but due to the lack of individuals of adequate size in some sites only three individuals per species were considered. All selected individuals (N = 185) were in good health with no apparent crown or stem damage, a description of their characteristics is shown in Supplementary Table 1. The field work complied with all local and national regulations and permission were obtained to visit all study sites. The used plant material was sampled under scientific collection license Flor-0154. Identification of plant material in field was carried out by Dr. Mirna Valdez-Hernández and no herbarium vouchers were collected or deposited.

### Environmental parameters

The total precipitation and pan evaporation, as well as the average environmental temperature at each site, were obtained from the meteorological stations of the National Water Commission. Stations were located 5–12 km away from the sites.

### Standardized precipitation index

The standardized precipitation index, developed by McKee^67^, is a powerful, flexible index that is simple to calculate and capable to define the main features of the meteorological drought, such as lead-time, duration, severity, magnitude and intensity based on the values on different time scales^68^. The drought index allows find the intensity of the drought among sites and periods and has been used to correlate the plant responses to drought^15^. In this study, standardized precipitation index was calculated monthly for the years 2015, 2016 and 2017. The index was calculated as the difference between a precipitation value of a period and the historical mean value (30 years, 1984–2014) given for the same period, which is then divided by the standard deviation of the historical mean value^67^. Standardized precipitation index values above 0 indicate a wet period, a value of ≤ − 1 indicates mild drought, values between − 1.0 and − 1.49 indicate moderate drought, values − 1.50 to − 1.99 show severe drought, and values ≤ − 2.0 indicate extreme drought.

### Functional traits

The functional traits characterized were wood density, wood relative water content, xylem water potential, and specific leaf area. Measurements were made in the rainy season (October) in three consecutive years: 2015 (pre-ENSO conditions), 2016 (ENSO conditions) and 2017 (post-ENSO conditions). During this period, we aimed to observe the ENSO effect due to the increase of the *canícula*^3^. In all cases, samples were taken on consecutive days during 2–3 days per site.

### Wood density and wood relative water content

The wood density and wood relative water content were obtained from wood samples taken at a height of 1.3 m from four individuals per species and site. Samples were taken with a core borer (5 mm inner diameter), sapwood was separated from heartwood, and the cores were placed in hermetic tubes and transported in a container with ice to avoid dehydration. Afterwards (in less than 12 h), length of each wood sample was measured and weight was achieved with an analytical balance (PA214C, OHAUS; Parsippany, NJ, USA) to obtain the fresh weight, samples were then placed for 48 h in distilled water and weighed again to obtain the saturated weight. Samples were then dried in an oven at 80 °C for 72 h and weighed to obtain the dry weight^62^. Wood density (g cm^−3^) was attained as the ratio of the dry weight of the sample over its volume, using sample length and core borer diameter (5 mm) to calculate the volume of a cylinder^18^. With the same wood cores, wood relative water content (Eq. 1, %)^68^.

1 $$ wood\;relative\;water\;content = \frac{{\left( {fresh\;weight - dry\;weight} \right)}}{{\left( {saturated\;weight - dry\;weight} \right)}} \times 100 $$

### Xylem water potential

Xylem water potential was determined in four individuals per species, per category, and per site. Measurements were performed with a pressure chamber (Model 1505D, PMS Instrument Company; Oregon, USA) on three terminal branches. Branches were collected at predawn (04–06 h), and at midday (12–14 h). Samples were placed in hermetic bags and stored in a cooler to avoid dehydration. All samples were measured within a maximum period of 2 h^18^.

### Specific leaf area

Specific leaf area was obtained from ten leaves completely exposed and ten shaded leaves to obtain an average for individual (four individuals per species). All collected leaves were photographed to determine the leaf area with the Imagej software ver. 1.48^69^. Leaves were then dehydrated in a drying oven at 80 °C for 48 h. An analytical balance was used to obtain the dry weight (PA214C, OHAUS), and specific leaf area was obtained dividing leaf area by leaf dry weight.

### Statistical analysis

The differences in each environmental parameter (precipitation, temperature and evaporation) for each site were tested by a one-way analysis of variance (ANOVA), considering year as factor.

To test the impact of ENSO according to leaf pattern and water availability at the sites, we used a mixed model ANOVA with fixed and random factors in which all individuals were included without considering the diameter category. Sampling leaf patterns (deciduous and evergreen), years (2015 pre-ENSO, 2016 ENSO, 2017 post-ENSO) and sites (wet, intermediate, dry) were considered as fixed factors. Species were considered as random factors. All functional traits (wood density, relative water content, predawn and midday water potential, specific leaf area) were considered as response variables. To determine the impact of ENSO according to the size of the individuals, we made a mixed model ANOVA for each diametric category (young and mature). The factors were considered in the same way as the previous ANOVA. Prior to the statistical analysis, a homogeneity test of variances was performed on the data of the established parameters.

To establish the relationship of environmental parameters (precipitation, temperature, evaporation and standardized precipitation index) with functional traits (wood density, relative water content, specific leaf area, predawn and midday water potential), a Pearson correlation analysis was applied, in which four individuals per species were used in each diameter category considering the 3 years and sites. For the correlation, the considered environmental variables were accumulated precipitation, accumulated pan evaporation and average temperature, considering two periods of 15 days and 30 days before the sampling. The standardized precipitation index considered in the analysis was recorded in the month of October of each year. Furthermore, to perform the correlation we used each year series independently (2015 pre-ENSO, 2016 ENSO, 2017 post-ENSO) and sites (wet, intermediate, dry), since each one presents different environmental values considering a total of nine series. All analyses were performed with Statistica software ver. 12^70^.

## Supplementary Information

Supplementary Legends.
