# Supplementary material for: El Niño-Southern Oscillation affects the water relations of tree species in the Yucatan Peninsula, Mexico
Source: Sci Rep. 2021 May 17;11:10451. doi: 10.1038/s41598-021-89835-8 (PMC8129073; doi:10.1038/s41598-021-89835-8)
Supplement: Supplementary file 1 — Supplementary Legends. [file 41598_2021_89835_MOESM1_ESM.pdf]

El Niño-Southern Oscillation affects the water relations of tree species in the Yucatan Peninsula, Mexico

Jorge Palomo-Kumul[1], Mirna Valdez-Hernández[1, *], Gerald A. Islebe[1], Manuel J. Cach-Pérez[2], José Luis Andrade[3]

[1] El Colegio de la Frontera Sur Unidad Chetumal, Herbario, Chetumal, Quintana Roo, CP 77014, México.

[2] CONACYT - El Colegio de la Frontera Sur Unidad Villahermosa, Departamento de Agricultura, Sociedad y Ambiente, Villahermosa, Tabasco, CP 86280, México.

[3] Centro de Investigación Científica de Yucatán, Unidad de Recursos Naturales A.C., Mérida, Yucatán, CP 97205, México.

[*]Corresponding author. Email address: mavaldez@ecosur.mx, (M. Valdez-Hernández)

Supplementary Figure 1

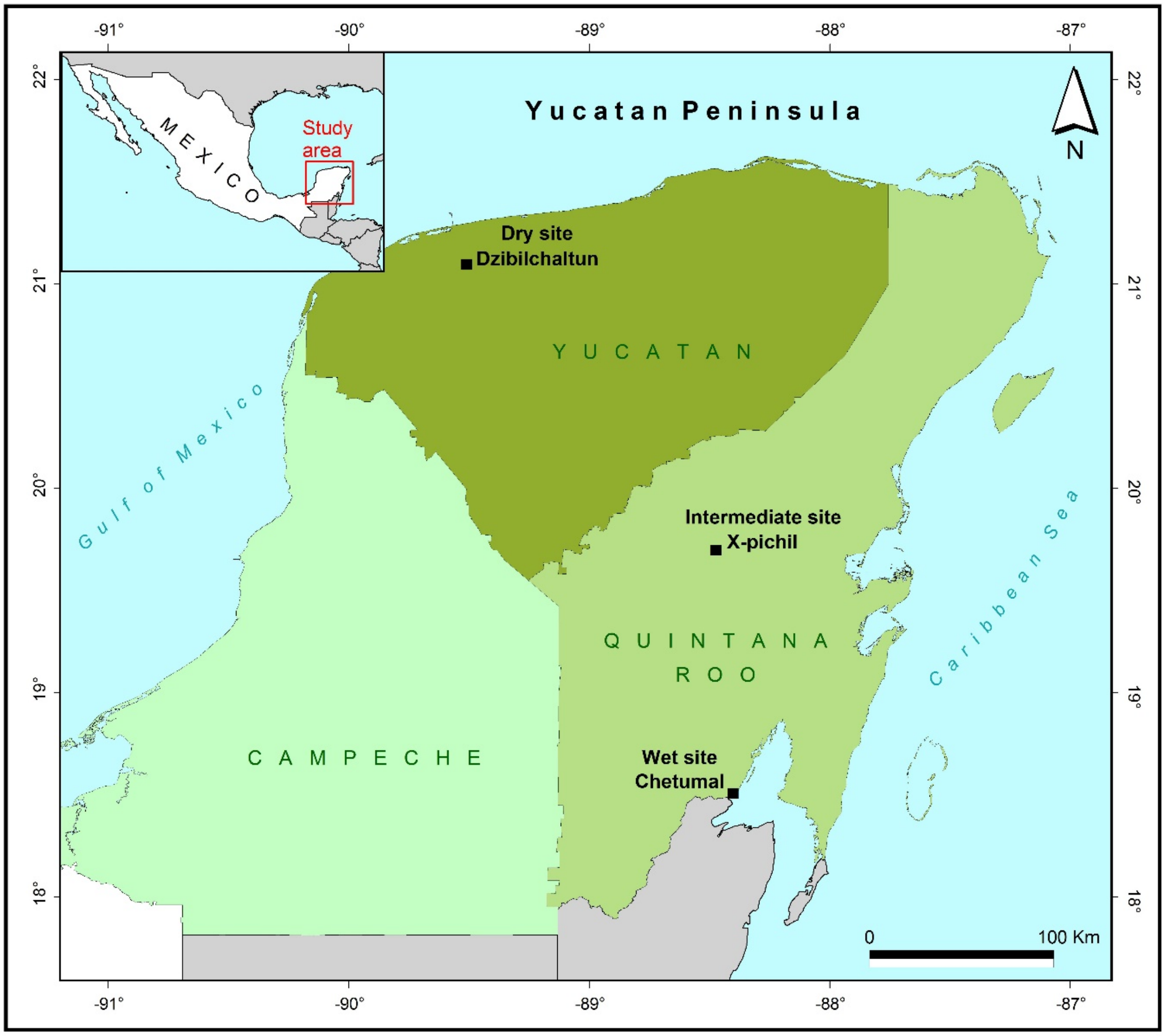

Supplementary Table 1.

Family	*Species*		*Individuals*			*DBH (cm)*		
			W	I	D	W	I	D
Evergreen species								
Malpighiaceae	*Byrsonima crassifolia*	Y	4	4	3	6.5±0.40	5.6±0.78	9.6±1.08
		M	4	4	3	29.2±3.85	20.7±0.45	24.5±1.53
Moraceae	*Brosimum alicastrum*	Y	4	4	4	8.7±0.86	9.9±0.40	9.5±0.27
		M	4	4	4	41.4±2.43	39.8±1.31	35.9±2.51
Sapotaceae	*Manilkara zapota*	Y	4	4	3	7.3±0.50	8.1±1.14	10.2±0.21
		M	4	4	3	34.9±3.16	24.8±0.94	33.3±0.98
	Chrysophyllum mexicanum	Y	4	4	3	9.9±0.69	9.1±1.10	8.9±1.97
		M	4	4	4	20.6±0.25	23.9±1.90	25.5±2.58
Deciduous species								
Boraginaceae	*Cordia dodecandra*	Y	4	4	3	9.8±0.60	8.4±1.86	8.9±0.16
		M	4	3	4	20.3±1.41	28.1±2.63	24.9±0.85
Fabaceae	*Piscida piscipula*	Y	4	4	4	8.9±0.47	7.2±0.55	8.6±1.30
		M	4	4	4	23.7±1.92	26.8±3.67	23.4±1.12
	Leucaena leucocephala	Y	4	4	4	5.7±0.19	5.2±0.47	6.1±0.60
		M	4	4	4	13.8±0.81	13.3±0.49	13.2±1.15
	Lysiloma latisiliquum	Y	4	4	4	9.7±0.31	5.9±0.43	6.7±0.42
		M	4	4	4	24.3±1.41	22.7±1.77	20.6±0.32

References

1. QGIS Development Team. QGIS Geographic Information System. Open Source Geospatial Foundation Project. http://qgis.osgeo.org (2018).
